# Implication of non-coding RNA-mediated ROCK1 regulation in various diseases

**DOI:** 10.3389/fmolb.2022.986722

**Published:** 2022-09-13

**Authors:** Soudeh Ghafouri-Fard, Yadollah Poornajaf, Bashdar Mahmud Hussen, Atefe Abak, Hamed Shoorei, Mohammad Taheri, Guive Sharifi

**Affiliations:** ^1^ Department of Medical Genetics, School of Medicine, Shahid Beheshti University of Medical Sciences, Tehran, Iran; ^2^ Faculty of Medicine, Birjand University of Medical Sciences, Birjand, Iran; ^3^ Department of Pharmacognosy, College of Pharmacy, Hawler Medical University, Erbil, Iraq; ^4^ Center of Research and Strategic Studies, Lebanese French University, Erbil, Iraq; ^5^ Men’s Health and Reproductive Health Research Center, Shahid Beheshti University of Medical Sciences, Tehran, Iran; ^6^ Clinical Research Development Unit of Tabriz Valiasr Hospital, Tabriz University of Medical Sciences, Tabriz, Iran; ^7^ Department of Anatomical Sciences, Faculty of Medicine, Birjand University of Medical Sciences, Birjand, Iran; ^8^ Institute of Human Genetics, Jena University Hospital, Jena, Germany; ^9^ Urology and Nephrology Research Center, Shahid Beheshti University of Medical Sciences, Tehran, Iran; ^10^ Skull Base Research Center, Loghman Hakim Hospital, Shahid Beheshti University of Medical Sciences, Tehran, Iran

**Keywords:** miRNA, lncRNA, ROCK1, expression, biomarker

## Abstract

Rho Associated Coiled-Coil Containing Protein Kinase 1 (ROCK1) is a protein serine/threonine kinase which is activated upon binding with the GTP-bound form of Rho. This protein can modulate actin-myosin contraction and stability. Moreover, it has a crucial role in the regulation of cell polarity. Therefore, it participates in modulation of cell morphology, regulation of expression of genes, cell proliferation and differentiation, apoptotic processes as well as oncogenic processes. Recent studies have highlighted interactions between ROCK1 and several non-coding RNAs, namely microRNAs, circular RNAs and long non-coding RNAs. Such interactions can be a target of medications. In fact, it seems that the interactions are implicated in therapeutic response to several medications. In the current review, we aimed to explain the impact of these interactions in the pathoetiology of cancers as well as non-malignant disorders.

## Introduction

*Rho Associated Coiled-Coil Containing Protein Kinase 1* (*ROCK1*) human gene is located on 18q11.1 The protein serine/threonine kinase encoded by this gene is activated upon binding with the GTP-bound form of Rho. Functioning as a small GTPase, Rho can regulate construction of focal adhesion molecules and stress fibers in fibroblasts, establishment of adhesion molecules that induce platelet aggregation and lymphocyte adhesion. Activity of Rho is regulated through binding with GDP or GTP. ROCK1 is regarded as an important modulator of actin-myosin contraction and stability. Moreover, it has a crucial role in the regulation of cell polarity. Therefore, it participates in modulation of cell morphology, regulation of expression of genes, cell proliferation and differentiation, apoptotic processes as well as stemness and oncogenic processes (Rath and Olson, 2012).

In fact, members of the Rho family such as RhoA and RhoC can enhance production of actomyosin contractile force *via* ROCK1- and ROCK2-mediated phosphorylation of several downstream targets, such as LIMK1/2 and MLC (Riento and Ridley, 2003). ROCK proteins have catalytic kinase domain responsible for the substrate promiscuity, a coiled-coil region, and a split PH domain that is intersected by the protein kinase C conserved region 1 (Rath and Olson, 2012). A single Rho-binding domain (RBD) exists inside the coiled-coil region of both ROCK proteins (Fujisawa et al., 1996), in addition to several Rho GTPases-interacting regions which have been identified within the coiled-coil region of ROCK1, which contributes to its localization (Blumenstein and Ahmadian, 2004).

**FIGURE 1:**
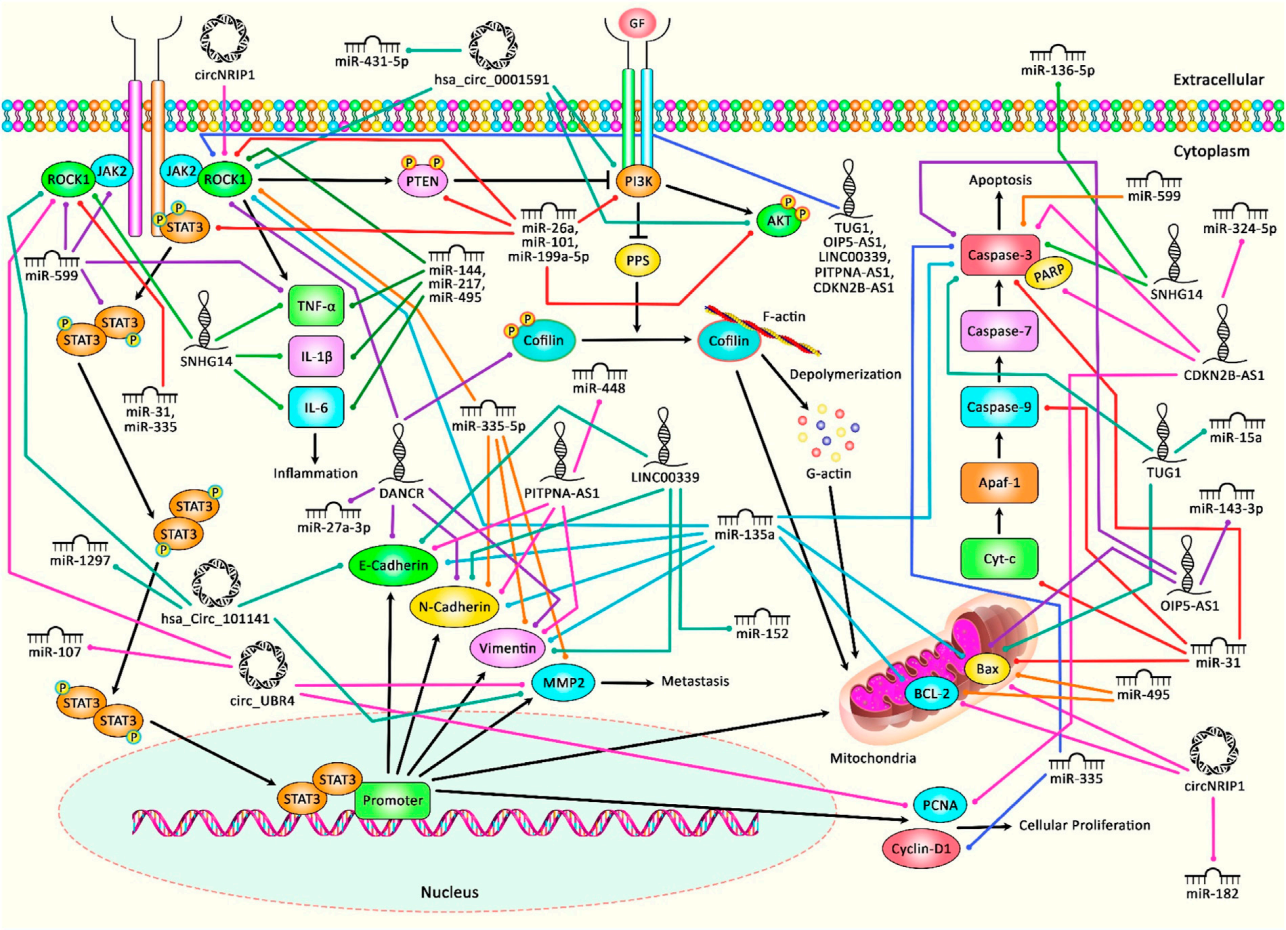
A schematic diagram of the role of several ncRNAs in triggering the ROCK1 signaling pathway in human disorders and malignancies. Overexpression of ROCK1 could result in triggering the activation of PTEN and PI3K, leading to PP1 and PP2A upregulation, and dephosphorylation of cofilin that could bind to G-actin and translocate to mitochondria, and eventually could cause cytochrome c release, caspases activation and apoptosis. ROCK1 could also play an effective role in activating and rapidly phosphorylating JAK2, which in turn could enhance downstream signaling cascades containing STAT3 and PI3K. Previous studies have authenticated that several ncRNAs (miRNAs, circRNAs, and lncRNAs) could have a crucial role in regulating the ROCK1 pathway in various human diseases as well as cancers. All the information regarding the role of these ncRNAs in the modulation of this cascade can be seen in Tables 1–7.

Recent studies have highlighted interactions between ROCK1 and several non-coding RNAs, namely microRNAs (miRNAs), circular RNAs (circRNAs) and long non-coding RNAs (lncRNAs). In the current review, we aimed to explain the impact of these interactions in the pathoetiology of cancers as well as non-malignant disorders. Figure 1 illustrates that aberrant expression of various ncRNAs could contribute in adversely modulating the ROCK1 pathway, with consequent triggering several kinds of cancers as well as a number of non-malignant conditions.

### ROCK1-interacting microRNAs in non-malignant conditions

Interactions between miRNAs and ROCK1 have been assessed in different disorders, including metabolic syndrome, diabetes, acute lung injury, endometriosis, LPS-induced lung endothelial hyperpermeability and pneumonia. These miRNAs mainly bind to 3′ UTR of ROCK and suppress its expression. Thus, the underlying mechanisms of such interactions are shared between these disorders. For instance, Guo et al. showed up-regulation of levels of a ROCK1-targeting miRNA, namely miR-324-5p, in the circulation of patients with hyperglycemia or hyperlipidemia. Investigations in an animal model of diabetes type II and obesity also verified over-expression of miR-324-5p both in the peripheral blood and hepatic tissue. Up-regulation of this miRNA results in reduction of activity of the AKT/GSK pathway and enhancement of lipid buildup. Moreover, ROCK1 silencing has resulted in deterioration of lipid and glucose metabolism. Notably, ROCK1 silencing has overturned the effect of miR-324-5p inhibition on amelioration of glucose and lipid metabolism. Taken together, miR-324-5p was shown to regulate metabolism of glucose and lipid through influencing expression of ROCK1 (Guo et al., 2020). Another miRNA, miR-217, was shown to affect immune responses and proliferative and migratory potential of vascular smooth muscle cells (VSMCs) in high-glucose condition through modulation of ROCK1. Expression of miR-217 was increased in high glucose-exposed VSMCs as well as aorta VSMCs obtained from diabetic animals. Mechanistically, miR-217 can induce cell cycle arrest, inhibit of proliferation, reduce migration, and enhance apoptosis of VSMCs in high glucose conditions through regulation of expression of ROCK1 (Zhou et al., 2021).

Another experiment in an animal model of sepsis-induced acute lung injury demonstrated the effect of miR-539-5p in alleviation of lung injury through modulation of expression of ROCK1. miR-539-5p could also decrease apoptotic potential and inflammatory responses in LPS-treated pulmonary microvascular endothelial cells of mice. The effects of miR-539-5p in inhibition of caspase-3 activity and inhibition of release of inflammatory cytokines have been reversed by up-regulation of ROCK1 (Meng et al., 2019).

Another study revealed the down-regulation of miR-202-3p expression in primary endometrial stromal cells obtained from eutopic or ectopic endometriosis compared to endometrial stromal cells from normal endometrium. Functional studies have shown that up-regulation of miR-202-3p impairs viability, migratory potential, and invasion of these cells, while it is silencing has the opposite impact. miR-202-3p mimics could decrease expression of ROCK1 in endometrial stromal cells. Taken together, dysregulation of miR-202-3p can participate in the pathogenesis of endometriosis through influencing expression of ROCK1 (Zhang et al., 2020a). Table 1 indicates the role of ROCK1-interacting miRNAs in non-malignant disorders.

**TABLE 1:** ROCK1-interacting miRNAs in non-malignant conditions (ALI: acute lung injury, LEHP: LPS-induced lung endothelial hyperpermeability).

Type of diseases	miRNA/expression pattern	Sample	Cell line	Target/Pathway	Molecular mechanism	References
Metabolic Syndrome	miR-324-5p (Up)	Peripheral blood samples: hyperglycemia (*n* = 102), hyperlipidemia (*n* = 106), healthy control (*n* = 110); db/db and C57BL/6 J mice	HepG2	ROCK1, AKT, GSK, PEPCK, FAS, ACC	Enhancing peripheral blood miR-324-5p by suppressing ROCK1 could promote the risk of metabolic syndrome	Guo et al. (2020)
Diabetes	miR‐217 (Down)	SD rats	VSMCs	ROCK1, TNF-α, IL-6, IL-1β	Up-regulation of miR‐217 could alleviate high‐glucose‐induced VSMCs dysfunction *via* targeting ROCK1	Zhou et al. (2021)
ALI	miR-539-5p (Down)	male C57BL/6 mice	MPVECs	ROCK1	miR-539-5p could alleviate sepsis-induced ALI by targeting ROCK1	Meng et al. (2019)
Endometriosis	miR-202-3p (Down)	Endometriosis patients (*n* = 27), health control (*n* = 31)	ESCs	ROCK1	Dysregulation of miR-202-3p could affect migration and invasion of ESCs in endometriosis *via* targeting ROCK1	Zhang et al. (2020a)
LEHP	miR-144 (-)	C57BL/6 J male mice	CC-3156, CC-4147	ROCK1, TNF-α, IL-1β	miR-144 could protect against LPS-induced LEHP *via* regulating ROCK1	Siddiqui et al. (2019)
Pneumonia	miR-495 (Down)	Pneumonia patients (*n* = 28), health control (*n* = 20)	293T, WI-38	ROCK1, Caspase-3, Bcl-2, Bax, IL-1β, IL-6, TNF-α	miR-495 could inhibit LPS-induced WI-38 cells apoptosis and inflammation by targeting ROCK1	Zhang et al. (2020b)
—	miR-599 (-)	—	HUVECs, 293T	ROCK1, JAK2, STAT3, TNF-α, Caspase-3, p53	miR-599 could regulate LPS-mediated apoptosis and inflammatory responses of HUVECs by targeting ROCK1	Wang et al. (2020a)
—	miR-135a (Down)	SD rats	TSPCs, 293T	ROCK1, p16	miR-135a could modulate tendon stem/progenitor cell senescence *via* suppressing ROCK1	Chen et al. (2015)

### ROCK1-interacting microRNAs in cancers

Similarly, cancer-related miRNAs can bind to 3′ UTR of ROCK1 to regulate its expression. A number of ROCK1-interacting miRNAs have been found to reduce tumor burden. For instance, experiments in non-small cell lung carcinoma cells showed that the tumor suppressor roles of miR-135a (Zhao et al., 2020), miR-148b (Luo and Liang, 2018) and miR-335-5p (Du et al., 2019) are exerted through modulation of expression of ROCK1. The interactions between miRNAs and ROCK1 have been mostly assessed in osteosarcoma cells among other cancers. miR-101 (Jiang et al., 2017), miR-139 (Fan et al., 2019), miR-144 (Liu et al., 2019), miR-202-5p (Li et al., 2018), miR-150 (Li et al., 2017a), miR-335 (Wang et al., 2017) and miR-214-5p (Zhang et al., 2017) are examples of down-regulated miRNAs in this type of cancer that were shown to directly regulate expression of ROCK1.

Roberto et al. measured expression of a number of ROCK1/ROCK2-targeting miRNAs, namely miR-124-3p, miR-138-5p, miR-139-5p, miR-335-5p and miR-584-5p in samples obtained from patients with Ewing sarcoma. They reported down-regulation of ROCK1 in these tissues; however its expression has not been associated with pathological factors. Expression levels of miR-124-3p, miR-139-5p and miR-335-3p were also shown to be reduced in these samples in correlation with ROCK1 levels. Down-regulation of miR-139-5p and miR-584-5p has been associated with disease progression. Moreover, down-regulation of miR-139-5p and miR-124-3p has been linked with poor clinical outcome. However, the results of *in vitro* studies on function of miR-139-5p were inconsistent. While its overexpression has led to a significant decrease in invasive abilities of cells, their clonogenic capability was enhanced (Roberto et al., 2020).

Expression levels of ROCK1-targeting miR-592 were reported to be decreased in clinical samples from patients with acute myeloid leukemia (AML) as well as AML cell lines. Down-regulation of miR-592 was associated with advanced French-American-British classification and adverse clinical outcomes. Functional studies also showed that up-regulation of miR-592 inhibits cell growth and metastatic capacity of cells, and enhances apoptosis (Xu et al., 2019). Table 2 shows the role of ROCK1-interacting miRNAs in cancers.

**TABLE 2:** ROCK1-interacting miRNAs in cancers (ANTs: adjacent non-cancerous tissues, NSCLC: non-small cell lung cancer, OS: osteosarcoma, EWS: Ewing sarcoma, AML/CML: acute/chronic myeloid leukemia, HCC: hepatocellular carcinoma, CRC: colorectal cancer).

Type of cancer	miRNA/expression pattern	Sample	Cell line	Target/Pathway	Molecular mechanism	References
NSCLC	miR-135a (Down)	NSCLC patients (*n* = 60)	HCC366, HCC827, NCI-H524, MRC-5, NCI-H1770	ROCK1, Bax, Bcl-2, Caspase-3, Vimentin, E/N-cadherin	miR-135a could inhibit malignant proliferation and diffusion of NSCLC by down-regulation of ROCK1 protein	Zhao et al. (2020)
NSCLC	miR-148b (Down)	16 pairs of NSCLC and ANTs	HBE1, H1299, H1650, H460, A549	ROCK1	miR-148b by regulating ROCK1 could inhibit proliferation and increase radiosensitivity of NSCLC.	Luo and Liang, (2018)
NSCLC	miR-335-5p (Down)	NSCLC tissue samples (*n* = 60)	16HBE, A549, HCC827, H1299, H1975, SPC-A1, H226, H1650, H460	ROCK1, TGF-β1, N-cadherin, Snail, Vimentin, MMP2	miR-335-5p *via* targeting ROCK1 can inhibit TGF-β1-induced EMT in NSCLC.	Du et al. (2019)
OS	miR-101 (Down)	20 pairs of OS and ANTs	MG63, U2OS, OS732, hFOB1.19	ROCK1, PTEN, JAK1, STAT3, PI3K/AKT	miR-101 can inhibit proliferation, invasion, and migration and in OS cells by targeting ROCK1	Jiang et al. (2017)
OS	miR-139 (Down)	OS (*n* = 25), non-tumor tissue samples (*n* = 19)	HOS, SAOS2, MG-63, U2OS, OS732, hFOB1.19	ROCK1, β-catenin, E-Cadherin, p53	miR-139 by targeting ROCK1 could inhibit OS cell proliferation and invasion	Fan et al. (2019)
OS	miR-144 (Down)	51 pairs of OS and ANTs	hFOB1.19	ROCK1, RhoA	miR-144 could inhibit tumor growth and metastasis in OS *via* dual-suppressing the RhoA/ROCK1 axis	Liu et al. (2019)
OS	miR-202-5p (Down)	36 pairs of OS and ANTs	U2OS, MG-63, HOS, hFOB1.19	ROCK1	miR-202-5p could inhibit the migration and invasion of OS cells by targeting ROCK1	Li et al. (2018)
OS	miR-150 (Down)	40 pairs of OS and ANTs	e SaOS2, U2OS, MG63, hFOB1.19	ROCK1	miR-150 could suppress cell proliferation, migration, and invasion of OS by targeting ROCK1	Li et al. (2017a)
OS	miR-335 (Down)	OS (*n* = 91), non- tumor tissue samples (*n* = 47)	-	ROCK1	miR-335 could influence tumor progression and prognosis of this cancer by targeting ROCK1	Wang et al. (2017)
OS	miR-214-5p (Down)	48 pairs of OS and ANTs	hFOB, HOS, MG63, G293, SAOS2, U2OS	ROCK1	miR-214-5p can suppress proliferation and invasion of OS cells by targeting ROCK1	Zhang et al. (2017)
EWS	miR-124a-3p, miR139-5p, miR-584-5p; (Down)	19 pairs of melanoma and adjacent normal tissues	SK-ES-1, RD-ES	ROCK1	Dysregulation of microRNAs could contribute to tumor progression of EWS by targeting ROCK1	Roberto et al. (2020)
AML	miR-592 (Down)	94 pairs of AML and ANTs	HS-5, HL-60, THP-1, NB4	ROCK1, MTHFD2	miR-592 could function as a tumor suppressor in AML by targeting ROCK1	Xu et al. (2019)
CML	miR-497-5p (Down)	Peripheral blood samples of CML patients (n = 57) and normal control group (n = 50)	K562, NHL	ROCK1	miR-497-5p could induce apoptosis in K562 cells by down-regulation of ROCK1	Chen et al. (2021a)
CRC	miR-199a-5p (Down)	40 pairs of CRC and ANTs; nude mice	SW480, HT29, LoVo, LS174T, SW620, HCT116, NCM460	ROCK1, STAT3, PI3K/AKT	miR-199a-5p could inhibit the growth and metastasis of CRC by targeting ROCK1	Zhu et al. (2018)
HCC	miR-145 (Down)	9 pairs of HCC and ANTs	HepG2	ROCK1, NF-κB, CCNE1	miR-145 could inhibit proliferation and increase apoptosis of HepG2 cells by targeting ROCK1	Pan et al. (2019)
HCC	miR-199a/b-5p (Down)	TCGA datasets, 35 pairs of HCC and ANTs; BALB/c nude mice	SMMC-7721, HepG2, Bel-7404, 97L, QSG-7701, 293T	ROCK1, MLC, ERK, PI3K/AKT	miR-199a/b-5p could inhibit hepatocellular carcinoma progression by post-transcriptionally suppressing ROCK1	Zhan et al. (2017)
HCC	miR-145 (Down)	96 pairs of HCC and ANTs	THLE-3, HepG2, Hep3B, PLC/PRF/5, MHCC97H	ROCK1	miR-145 could suppress cell proliferation and motility of HCC by inhibiting ROCK1	Ding et al. (2016)
Liver Cancer	miR-31 (Down)	-	HepG2, L02	ROCK1, Bax, Cyt-c, Caspase-3/9	miR-31 could modulate apoptosis and invasion of HepG2 cells *via* ROCK1/F-Actin axis	Zhang et al. (2020c)
Renal cell carcinoma	miR-199a (Down)	150 pairs of RCC and ANTs	ACHN, A498	ROCK1	miR-199a could affect the kidney cell invasion, proliferation, and apoptosis by targeting ROCK1	Qin et al. (2018)
Bladder cancer	miR-199a (Down)	98 pairs of RCC and ANTs; nude mice	A498	ROCK1	miR-199a, regulated by Snail, could modulate clear cell aggressiveness *via* repressing ROCK1	Zhang et al. (2018)
Bladder cancer	miR-335 (Down)	27 pairs of BLC and ANTs	T24, EJ	ROCK1	Down-regulation of miR-335 could enhance the invasion and migration of BLC cells *via* targeting ROCK1	Wu et al. (2016)
Breast cancer	miR-145 (Down)	88 pairs of BCa and adjacent normal tissues	MCF-7, BT-474, MDA-MB-453, BT-549, SK-BR-3, MDA-MB-231	ROCK1	miR-145 could inhibit the growth and migration of breast cancer cells *via* targeting oncoprotein ROCK1	Zheng et al. (2016)
Breast cancer	miR-106b-5p (Down)	GEO database, 20 pairs of BCa and adjacent normal tissues	MCF-10A, MCF-7, MDA-MB-231, 293T, CAMA-1, T47D	ROCK1, Rho, CNN1, STAT1	miR-106b-5p could contribute to the lung metastasis of BCa *via* targeting CNN1 and regulating Rho/ROCK1 axis	Wang et al. (2020b)
Thyroid cancer	miR-26a (Down)	51 pairs of PTC and adjacent normal	BCPAP, TPC-1, K1, HTH83	ROCK1, PI3K/AKT	miR-26a could suppress the malignant biological behaviors of PTC by targeting ROCK1 and regulating the PI3K/AKT pathway	Wu et al. (2019)
Thyroid cancer	miR-584 (Down)	-	K1, TCP-1, W3	ROCK1	miR-584 could suppress invasion and cell migration of thyroid carcinoma by regulating ROCK1	Xiang et al. (2015)
GBM	miR-300 (Down)	Nude mice	U87, U373, U251, A172, NHAs	ROCK1	miR-300 by ROCK1 could inhibit GBM cells progression	Zhou et al. (2016)
Neuroblastoma	miR-506 (Down)	28 pairs of NB and ANTs	IMR-32, N2A, SK-N-SH, SH-SY5Y	ROCK1	miR-506 could suppress NB metastasis by targeting ROCK1	Li et al. (2017b)
Laryngeal squamous cell carcinoma	miR-195 (Down)	51 pairs of LSCC tissues and adjacent normal epithelial tissues	AMC-HN-8, Tu-177, Hep-2, HaCaT, 293T	ROCK1	miR-195 could inhibit cell proliferation, migration, and invasion of laryngeal squamous cell carcinoma by targeting ROCK1	Liu et al. (2017)
Melanoma	miR-335 (Down)	30 pairs of melanoma and adjacent normal tissues	A375, COLO829, HMCB PMWK, B16	ROCK1, Cyclin-D1, Caspase-3	miR-335 could act as a tumor suppressor and enhance ionizing radiation-induced tumor regression by targeting ROCK1	Cheng and Shen, (2020)

### ROCK1-interacting circular RNAs in non-malignant conditions

CircRNAs mainly affect expression of ROCK1 through sponging ROCK1-interacting miRNAs. These interactions have been assessed in the context of non-alcoholic fatty liver disease and atherosclerosis. Expression of circ_0057558 was shown to be increased in nonalcoholic fatty liver disease, parallel with down-regulation of miR-206. Circ_0057558 silencing and up-regulation of miR-206 could decrease accumulation of lipids and secretion of triglycerides. Functionally, miR-206 could directly target ROCK1 and activate AMPK pathway through this route. In fact, circ_0057558 serves as a miR-206 sponge to suppress AMPK signals. Cumulatively, circ_0057558/miR-206/ROCK1/AMPK was found to be a functional axis in the etiology of nonalcoholic fatty liver disease (Chen et al., 2021b).

Another study reported the up-regulation of circ_UBR4 in an *in vitro* model of atherosclerosis. Moreover, expression levels of circ_UBR4 and ROCK1 have been found to be increased in sera of patients with atherosclerosis, parallel with down-regulation of miR-107. Circ_UBR4 silencing has led to induction of cell cycle arrest, suppression of cell viability, colony-forming capability, migration aptitude, and depression of expression of proliferating cell nuclear antigen and MMP2. miR-107 was found to act as a mediator of circ_UBR4 effects on ROCK1 expression. Taken together, circ_UBR4/miR-107/ROCK1 pathway has a possible role in the development of atherosclerosis through modulation of proliferative ability, migration, and cell cycle transition of human VSMCs (Zhang et al., 2021). Table 3 shows the role of ROCK1-interacting circRNAs in non-malignant conditions.

**TABLE 3:** ROCK1-interacting circRNAs in non-malignant conditions (NAFTD: Non-alcoholic fatty liver disease, AS: atherosclerosis).

Type of diseases	CircRNA/expression pattern	Sample	Cell line	Interacting miRNA	Target/Pathway	Molecular mechanism	References
NAFLD	Circ_0057558 (Up)	C57BL/6 J mice	Huh-7, HepG2	miR-206	ROCK1, AMPK, CD-36, FAS, SCD1, ACC1, SREBP1	Circ_0057558 could promote non-alcoholic fatty liver disease *via* targeting miR-206 and regulating ROCK1/AMPK axis	Chen et al. (2021b)
AS	circ_UBR4 (Up)	Serum samples of AS patients (*n* = 41), healthy individuals (*n* = 41)	BNCC340087	miR-107	ROCK1, MMP2, PCNA	Circ_UBR4 could promote proliferation, migration, and cell cycle transition of human VSMCs in atherosclerosis	Zhang et al. (2021)

### ROCK1-interacting circular RNAs in cancers

A number of ROCK1-interacting circRNAs have been reported to be up-regulated in tissue or serum samples of patients with malignant conditions. For instance, circ-TIMELESS *via* the miR‐136‐5p/ROCK1 axis could regulate proliferation of lung squamous cell carcinoma cells (Zhang et al., 2020d). Moreover, hsa_circ_0001591 could promote metastasis and cell proliferation of human melanoma *via* modulation of ROCK1 through targeting miR-431-5p (Yin et al., 2021). hsa_circ_0043278 could promote cell proliferation and migration of NSCLC *via* sponging miR-520f and regulating ROCK1 expression (Cui et al., 2019). Finally, circ-ABCB10 could promote growth and metastasis of NPC by up-regulation of ROCK1 (Duan et al., 2020). Table 4 shows the role of ROCK1-interacting circRNAs in cancers.

**TABLE 4:** ROCK1-interacting circRNAs in cancers (ANT: adjacent non-cancerous tissue, LSCC: Lung squamous cell carcinoma, NSCLC: Non-small cell lung cancer, HCC: Hepatocellular carcinoma, GC: gastric cancer, RB: retinoblastoma, NPC: Nasopharyngeal carcinoma).

Type of cancer	CircRNA/expression pattern	Sample	Cell line	Interacting miRNAs	Target/Pathway	Molecular mechanism	References
LSCC	Circ-TIMELESS (hsa_circ_0000408) (Up)	45 pairs of LUSC and ANTs; BALB/c nude mice	NHBE, H520, H226	miR‐136‐5p	ROCK1	Circ-TIMELESS could regulate proliferation of lung squamous cell carcinoma cells *via* the miR‐136‐5p/ROCK1 axis	Zhang et al. (2020d)
Melanoma	hsa_circ_0001591 (Up)	Serum samples of M patients (*n* = 53) and health control (*N* = 53)	A2058	miR-431-5p	ROCK1, PI3K/AKT	hsa_circ_0001591 could promote metastasis and cell proliferation of human melanoma by targeting miR-431-5p	Yin et al. (2021)
NSCLC	hsa_circ_0043278 (Up)	44 pairs of NSCLC and adjacent normal; Male BALB/c mice	16HBE, H1975, A549, SPC-A1, H1299	miR-520f	ROCK1, CDKN1B	hsa_circ_0043278 could promote cell proliferation and migration of NSCLC *via* sponging miR-520f and regulating ROCK1	Cui et al. (2019)
HCC	hsa_Circ_101141 (Up)	60 pairs of NSCLC and ANTs	HCCLM3, 293T, SK-HEP-1, Hep3B, Huh7, LO2	miR-1297	ROCK1, MMP2, E-cadherin, p21, cylin-D1	hsa_Circ_101141 could facilitate tumorigenesis of hepatocellular carcinoma by regulating the miR-1297/ROCK1 axis	Zhang et al. (2020e)
HCC	Circ_0009910 (Up)	28 pairs of HCC and ANTs; male nude mice	HepG2, 293T, HCCLM3, L02, MHCC97L	miR-335-5p	ROCK1	Circ_0009910 could promote proliferation and metastasis of HCC *via* the miR-335-5p/ROCK1 axis	Pegoraro et al. (2020)
GC	circNRIP1 (Up)	45 pairs of GC and ANTs	MGC-803, AGS, HGC-27, GES-1	miR-182	ROCK1, Bcl 2, Bax	CircNRIP1 could promote cell apoptosis by regulating miR-182/ROCK1 axis	Liang and Li, (2020)
RB	Circ_E2F3 (Up)	23 RB tissues and 16 normal retina tissues	ARPE-19, Y79, SO-RB50, WERI-RB-1	miR-204-5p	ROCK1	Circ-E2F3 could promote proliferation and metastasis of retinoblastoma *via* the miR-204-5p/ROCK1 axis	Huang et al. (2021)
NPC	Circ_ABCB10 (Up)	45 pairs of NPC and ANTs	CNE2, 5-8F, 6-18B, NP69	-	ROCK1	Circ-ABCB10 could promote growth and metastasis of NPC by up-regulation ofROCK1	Duan et al. (2020)

### ROCK1-interacting long non-coding RNAs in non-malignant conditions

Similar to circRNAs, lncRNAs can act as sponges for ROCK1-interacting miRNAs. Experiments in an animal model of Alzheimer’s disease confirmed reduction of spatial learning and memory abilities, noticeable pathological injuries, increase in apoptosis of hippocampal neurons and reduction of antioxidant ability. TUG1 silencing and miR-15a up-regulation could result in improvement of spatial learning and memory capacities, amelioration of pathological injuries, suppression of apoptosis of neurons, and enhancement of antioxidant capacity of hippocampal neurons in the animal model of Alzheimer’s disease. *In vitro* studies have also confirmed that TUG1 silencing and miR-15a up-regulation constrains apoptosis of hippocampal neurons. This miRNA directly targets ROCK1 (Li et al., 2020). Another study has shown that SNHG14 can assist in induction of inflammatory response by cerebral ischemia/reperfusion (I/R) injury *via* regulating miR-136-5p/ROCK1 axis (Zhong et al., 2019). SNHG7 is another ROCK1-interacting lncRNA which participates in the pathoetiology of cardiac fibrosis. Expression of this lncRNA was found to be up-regulated in the infarcted and peri-infarcted areas of animal models. SNHG7 silencing led to the reduction of expression levels of Col1 and α-SMA. Moreover, suppression of SNHG7 levels resulted in improvement of cardiac function after myocardial infarction. SNHG7 acts as a molecular sponge for miR-34-5p. Co-transfection of SNHG7 and miR-34-5p suppressed viability and proliferative ability of cardiac fibroblasts. Taken together, SNHG7 has a role in induction of cardiac fibrosis through modulation of miR-34-5p/ROCK1 axis (Wang et al., 2020c). Table 5 shows the role of ROCK1-interacting lncRNAs in non-malignant conditions.

**TABLE 5:** ROCK1-interacting lncRNAs in non-malignant conditions (AD: Alzheimer’s disease, Cerebral I/R injury: Cerebral ischemia/reperfusion injury, CF: Cardiac fibrosis, NAFLD: Non-alcoholic fatty liver disorder, OP: Osteoporosis).

Type of diseases	lncRNA/expression pattern	Sample	Cell line	Interacting miRNAs	Target/Pathway	Molecular mechanism	References
AD	TUG1 (Down)	BALB/c mice	Hippocampal Neurons (HN)	miR-15a	ROCK1, Bax, Caspase-3	Knockdown of TUG1 could depress apoptosis of hippocampal neurons by elevating miR-15a and repressing ROCK1	Li et al. (2020)
Cerebral I/R injury	SNHG14 (Up)	SD rats	PC-12	miR-136-5p	ROCK1, Caspase-3, IL-1β, IL-6, TNF-α	SNHG14 promotes inflammatory responses induced by cerebral I/R injury *via* regulating miR-136-5p/ROCK1 axis	Zhong et al. (2019)
CF	SNHG7 (Up)	C57BL/6 mice	-	miR-34-5p	ROCK1, TGF-β1	SNHG7 could promote cardiac remodeling *via* sponging miR-34-5p and up-regulation of ROCK1	Wang et al. (2020c)
NAFLD	NEAT1 (Up)	C57BL/6 J mice	HepG2	miR-146a-5p	ROCK1, SREBP1c, FAS, ACC, CPT1	NEAT1 could promote hepatic lipid accumulation in NAFLD *via* regulating miR-146a-5p/ROCK1 axis	Chen et al. (2019)
OP	ROR (Down)	Affected persons (*n* = 82), healthy controls (*n* = 79)	MC3T3-E1	miR-145-5p	ROCK1	LncRNA ROR could modulate the osteoblasts proliferation and apoptosis by regulating miR-145-5p/ROCK1 axis	Fu et al. (2021)

### ROCK1-interacting long non-coding RNAs in cancers

The impact of ROCK1-interacting lncRNAs on carcinogenesis has been evaluated in different cancers such as lung cancer, osteosarcoma, hepatocellular carcinoma and cervical cancer. For instance, PSMG3-AS1 *via* down-regulation of miR-340 and subsequent up-regulation of ROCK1 could promote cell migration and invasion of non-small cell lung carcinoma (Wang et al., 2021a). Moreover, KCNMB2-AS1 *via* sponging miR-374a-3p and regulating ROCK1 could assist in the progression of lung cancer (Yang et al., 2020).

In osteosarcoma, HAGLROS could promote cell invasion and metastasis *via* sponging miR-152 and up-regulation of ROCK1 (Zhou et al., 2020). Moreover, DANCR could promote proliferation and metastasis of these cells *via* sponging ROCK1-targeting miRNAs miR-335-5p and miR-1972 (Wang et al., 2018). Finally, HOXA11-AS could enhance the invasion and migration of osteosarcoma *via* sponging miR-124-3p and up-regulation of ROCK1 (Cui et al., 2017).

In cervical cancer, OIP5-AS1 (Song et al., 2020) and DANCR (Liang et al., 2019) were found to up-regulate ROCK1 *via* sponging miR-143-3p and miR‐335‐5p, respectively. Table 6 shows the role of ROCK1-interacting lncRNAs in cancers.

**TABLE 6:** ROCK1-interacting lncRNAs in cancers (ANT: adjacent non-cancerous tissue, NSCLC: non-small cell lung cancer, OS: osteosarcoma, HCC: hepatocellular carcinoma, ESCC: Esophageal squamous cell carcinoma, CC: cervical cancer, OC: ovarian cancer, BCa: breast cancer, LSCC: Laryngeal squamous cell carcinoma).

Type of cancer	lncRNA/expression pattern	Sample	Cell line	Interacting miRNAs	Target/Pathway	Molecular mechanism	References
NSCLC	PSMG3-AS1 (Up)	60 pairs of NSCLC and ANTs	H1993	miR-340	ROCK1	PSMG3-AS1 could promote cell migration and invasion *via* down-regulation of miR-340 and up-regulation of ROCK1	Wang et al. (2021a)
NSCLC	KCNMB2-AS1 (Up)	61 pairs of NSCLC tissues and ANTs	A549, SK-MES-1, BEAS-2B, H522, H460	miR-374aa-3p	ROCK1	KCNMB2-AS1 *via* sponging miR-374a-3p and regulating ROCK1 could facilitate the progression of NSCLC.	Yang et al. (2020)
SCLC	MCM3AP-AS1 (Up)	60 pairs SCLC of and ANTs	SHP-77	miR-148a	ROCK1	MCM3AP-AS1 could enhance cell invasion and migration of small cell lung carcinoma *via* sponging miR-148a and elevating ROCK1	Luo et al. (2021)
NSCLC	KCNMB2-AS1 (Up)	61 pairs of SCLC and ANTs	A549, SK-MES-1, H460, BEAS-2B	miR-374a-3p	ROCK1	KCNMB2-AS1 could facilitate the progression of NSCLC *via* sponging miR-374a-3p and increasing ROCK1 expression	Yang et al. (2020)
OS	HAGLROS (Up)	10 pairs of OS and ANTs	MG-63, hFOB 1.19, SW1353, U2OS	miR-152	ROCK1	HAGLROS could promote cell invasion and metastasis of osteosarcoma *via* sponging miR-152 and up-regulation of ROCK1	Zhou et al. (2020)
OS	DANCR (Up)	95 pairs of OS and ANTs; Female nude mice	MG-63, U2OS, MNNG/HOS, 143B, hFOB 1.19	miR-335-5p, miR-1972	ROCK1	DANCR could promote proliferation and metastasis of OS cells *via* sequestering miR-335-5p and miR-1972	Wang et al. (2018)
OS	HOXA11-AS (Up)	51 pairs of OS and ANTs; nude mice	U2OS, MG-63, KHOS, NHost	miR-124-3p	ROCK1	HOXA11-AS could enhance the invasion and migration of OS cells *via* sponging miR-124-3p	Cui et al. (2017)
HCC	DANCR (Up)	Databases; BALB/C nude mice	L02, Hep3B, Huh7, HepG2, MHCC‐97H, HCC‐LM3	miR‐27a‐3p	ROCK1, LIMK1, Cofilin-1, E/N-cadherin, Vimentin	DANCR could promote hepatocellular carcinoma progression *via* sponging miR‐27a‐3p and regulating the ROCK1/LIMK1/Cofilin-1 axis	Guo et al. (2019)
HCC	LINC00339 (Up)	60 pairs of HCC tissues and ANTs; BALB/c nude mice	L02, HUH7, HepG2, HUH‐6, SK‐Hep‐1, 293T	miR‐152	ROCK1, E-cadherin, N-cadherin, Vimentin	LINC00339 could enhance proliferation and migration of HCC *via* regulating miR‐152	Chen and Zhang, (2019)
HCC	PITPNA-AS1 (Up)	93 pairs of HCC tissues and ANTs; BALB/c female nude mice	L02, Hep3B, HepG2, HCCLM3, SMMC-7721	miR-448	ROCK1, E-cadherin, N-cadherin, Vimentin	PITPNA-AS1 could facilitate invasion and migration of HCC *via* the miR-448/ROCK1 axis	Wang et al. (2021b)
ESCC	EGFR-AS1 (Up)	56 pairs of ESCC tissues and ANTs	KYSE-30, EC109	miR-145	ROCK1	EGFR-AS1 could promote Invasion and Migration of ESCC *via* sponging miR-145 and up-regulation of ROCK1	Feng et al. (2020)
Liver Cancer	LINC00491 (Up)	TCGA, GEO databases	HUH-7, HepG2, HUH-6, SK-Hep-1	miR-324-5p	ROCK1	LINC00491 could promote cell growth and metastasis *via* miR-324-5p/ROCK1 axis	Wang et al. (2021c)
Pancreatic cancer	LINC00941 (Up)	54 pairs of PC and ANTs	AsPC-1, BxPC-3, PANC-1, Capan-2, HPDE	miR-335-5p	ROCK1, LIMK1, Cofilin-1, ZEB2, E/N-cadherin, Vimentin	LINC00941 promotes the progression of pancreatic cancer through binding with miR-335-5p and regulating the ROCK1-mediated LIMK1/Cofilin-1 axis	Wang et al. (2021d)
Leukemia	HOTAIRM1 (Up)	-	K562, U937, THP1, Jurkat, 293T, Kasumi-1, SKNO-1	-	ROCK1, RHOA, ARHGAP18, Bcl-2	HOTAIRM1 could enhance glucocorticoid resistance in leukemia by activating the RHOA/ROCK1 axis *via* suppressing ARHGAP18	Liang et al. (2021)
Glioma	LINC00346 (Up)	20 pairs of G and ANTs, BALB/c nude mice	NHAs, U87, H4, U251, LN229	miR-340-5p	ROCK1	LINC00346 could promote cell migration, invasion and proliferation of glioma cells by up-regulation of ROCK1	Qiu et al. (2020a)
CC	OIP5-AS1 (Up)	306 pairs of CC and ANTs	C33A	miR-143-3p	ROCK1, Bax, Caspase-3, Cyclin-A/B1	OIP5-AS1 in cervical cancer could affect expression of ROCK1 *via* sponging miR-143-3p	Song et al. (2020)
CC	DANCR (Up)	65 pairs of CV tissues and ANTs	Caski, SW756, SiHa, C33A, HeLa, ME‐180, End1/E6E7	miR‐335‐5p	ROCK1, E-cadherin, Vimentin	DANCR could promote CC progression *via* sponging miR‐335‐5p and up-regulation of ROCK1	Liang et al. (2019)
OC	SNHG20 (Up)	-	SKOV3, A2780, OVCAR-3, CAOV-3	miR-148a	ROCK1	SNHG20 could promote migration and invasion of ovarian cancer *via* modulating the miR-148a/ROCK1 axis	Yang and Dong, (2021)
BCa	PVT1 (Up)	BCa tissue samples (n = 30)	MCF-10, MCF7, MDA-MB-468, MDA-MB-231	miR-148a-3p	ROCK1	PVT1 could facilitate invasion and migration of breast cancer by regulating miR-148a-3p and ROCK1	Liu et al. (2021)
LSCC	CDKN2B-AS1 (Up)	60 pairs of LSCC tissues and ANTs	NP69, TU177, BNCC338439, BNCC341383, AMC-HN-8	miR-324-5p	ROCK1, PCNA, P21, Caspase-3, PARP	CDKN2B-AS1 could enhance invasion, migration, and proliferation of laryngeal squamous cell carcinoma *via* regulating miR-324-5p	Liu et al. (2020)

## The impact of interactions between non-coding RNAs and ROCK1 on therapeutic responses

**FIGURE 2:**
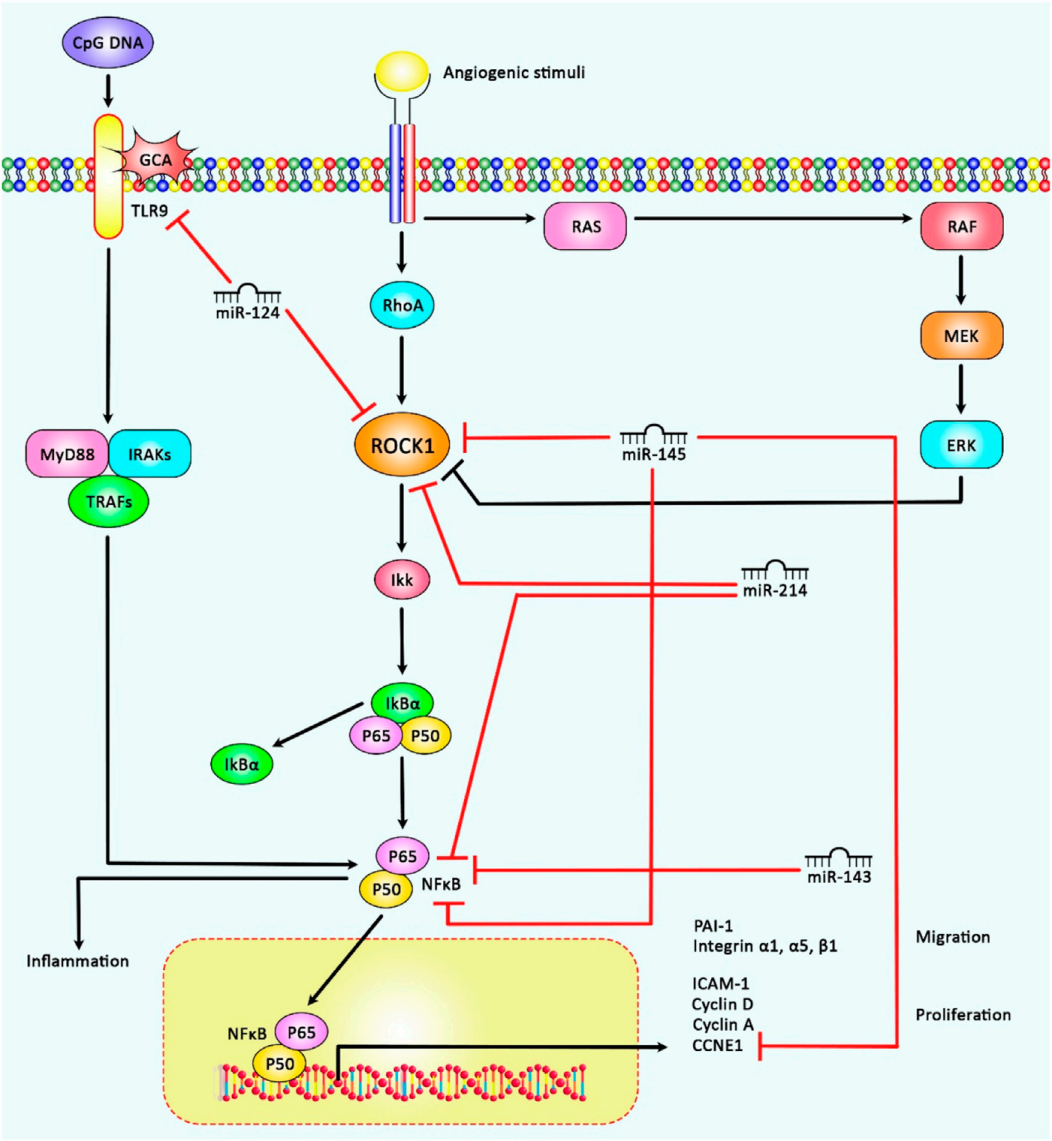
A schematic representation of the role of several miRNAs in regulating the ROCK1/NF-κB signaling cascade in cancers and non-malignant disorders. A recent study has detected that miR-145 could play a crucial role in inducing cell cycle suppression and activation of cell apoptosis, and thereby controlling hepatocellular carcinoma *via* down-regulation of the expression levels of ROCK1, NF-κB as well as CCNE1(27). Another research has demonstrated that up-regulation of miR-143 and miR-124 could down-regulate NF-kB and ROCK1 expression respectively, which could have a therapeutic role in Osteoarthritis (Qiu et al., 2020b). Moreover, accumulating evidence has represented that overexpression of ROCK1 could result in the activation of NF-κB that could in turn aggravate cerebral ischemia/reperfusion injury. Additionally, miR-214 *via* could target and negatively modulate ROCK1 and NF-κB expression, thereby could play a key role in the protection of DEX against cerebral ischemia/reperfusion injury (Liu et al., 2021)

A number of therapeutic agents have been found to act through regulation of ROCK1-interacting non-coding RNAs. For instance, sevoflurane through regulation of circ_0079593/miR-633/ROCK1 axis could suppress tumorigenesis process in glioma (Cheng and Cheng, 2021). In addition, dexmedetomidine (DEX) could ameliorate cerebral I/R injury *via* the miR-214/ROCK1/NF-κB axis (Liu et al., 2021). Besides, the therapeutic effects of curcumin in osteoarthritis are possibly exerted *via* modulating the miR-143/ROCK1/TLR9 and miR-124/NF-kB pathways (Qiu et al., 2020b). Furthermore, some ROCK1-interacting non-coding RNAs can affect response to therapeutic agents. For example, circ_PIP5K1A *via* regulation of miR-493-5p/ROCK1 axis could regulate cisplatin resistance in lung cancer (Feng et al., 2021). Moreover, miR-136-5p could enhance cisplatin sensitivity and suppress invasion and migration in head and neck cancer cells *via* targeting the ROCK1 (Yang et al., 2021). Table 7 shows the mutual interactions between drug and ROCK1-interacting non-coding RNAs. Figure 2 represents the role of several miRNAs in various human disorders *via* regulating the ROCK1/NF-κB signaling pathway.

**TABLE 7:** Drug and ROCK1-interacting non-coding RNAs (NSCLC: Non-small cell lung cancer, HNC: Head and neck cancer, Cerebral I/R injury: cerebral ischemia/reperfusion injury, HCC: hepatocellular carcinoma).

Type of diseases	Non-coding RNAs/expression pattern	Sample	Drug and dose	Cell line	Target/Pathway	Molecular mechanism	References
NSCLC	Circ_PIP5K1A (Up)	Tumor-sensitive (*n* = 33), tumor-resistant (*n* = 23); BALB/c male nude mice	Cisplatin, 0–30 μM; I.P, 6 mg/kg DDP once 2 days	A549, H460, A549/DDP, H460/DDP	ROCK1, miR-493-5p	Circ_PIP5K1A could regulate cisplatin resistance in NSCLC *via* regulation of miR-493-5p/ROCK1 axis	Feng et al. (2021)
HNC	miR-136-5p (-)	-	Cisplatin; 2.6 µM	FaDu, FD-LSC-1	ROCK1, E/N-cadherin, LC3II/I, Caspase-3, AKT/mTOR	miR-136-5p could enhance cisplatin sensitivity and suppress invasion and migration in head and neck cancer cells *via* targeting the ROCK1	Yang et al. (2021)
Cerebral I/R injury	miR-214 (-)	SD rats	Dexmedetomidine (DEX); intravenously, 1 μg/kg at the beginning of the surgery and 0.05 μg/kg/min for the next 2 h	-	ROCK1, NF-κB	DEX could ameliorate cerebral I/R injury *via* the miR-214/ROCK1/NF-κB axis	Liu et al. (2021)
HCC	miR-148a-3p (-)	ALB/c nude mice	Sevoflurane (SEVO); 1–8% SEVO mixed with 95% air and 5% CO2 at 6 L/min for 6 h, mice intravenously injected with 4% SEVO for 30 days	L02, Huh7, HCCLM3	ROCK1, p53, p21	miR-148a-3p could enhance the effect of SEVO on HCC progression *via* ROCK1 repression	Sun et al. (2021)
Glioma	Circ_0079593 (-)	Glioma patients (n = 34), normal brain tissues (n = 19); BALB/c nude mice	Cells treated with 0–5.1% SEVO for 6 h, mice subcutaneously injected with 5.1% SEVO for 7 days	T98G, LN-229, NHA	ROCK1, miR-633, E-cadherin, Vimentin	SEVO could suppress glioma tumorigenesis *via* regulating circ_0079593/miR-633/ROCK1 axis	Cheng and Cheng, (2021)
Osteoarthritis	miR-143, miR-124 (Down)	Mice	Curcumin; 1–5 μmol/L	BMSCs, primary chondrocytes	ROCK1, NF-κB, TLR9	Curcumin could reinforce BMSC-derived exosomes and attenuate osteoarthritis *via* modulating the miR-143/ROCK1/TLR9 and miR-124/NF-kB pathways	Qiu et al. (2020b)
Ischemia	miR-494-3p (Down)	SD rats	Ginsenoside Rg1; 100 μg/ml	rBMSCs	ROCK-1, MLC-2, Bax, Bcl-2	Ginsenoside can protect rBMSCs against ischemia-associated apoptosis Rg1 *via* the miR-494-3p and ROCK1	Zheng et al. (2018)

## Discussion

Several non-coding RNAs have been shown to interact with ROCK1. The interaction between ROCK1 and these transcripts can affect development of different types of cancers as well as a number of non-malignant conditions such as metabolic syndrome, diabetes, acute lung injury, pneumonia, endometriosis, non-alcoholic fatty liver disease, cerebral ischemia/reperfusion injury, myocardial Infarction, osteoporosis and atherosclerosis.

CircRNAs and lncRNAs that influence expression of ROCK1 mainly act through sponging ROCK1-targeting miRNAs. Circ_0057558/miR-206, circ_UBR4/miR-107, circ-TIMELESS/miR‐136‐5p, has_circ_0001591/miR-431-5p, hsa_circ_0043278/miR-520f, hsa_Circ_101141/miR-1297, Circ_0009910/miR-335-5p, circNRIP1/miR-182, circ_E2F3/miR-204-5p, TUG1/miR-15a, SNHG14/miR-136-5p, SNHG7/miR-34-5p, NEAT1/miR-146a-5p, lnc-ROR/miR-145-5p, PSMG3-AS1/miR-340, KCNMB2-AS1/miR-374aa-3p, MCM3AP-AS1/miR-148a, HAGLROS/miR-152, DANCR/miR-335-5p, DANCR/miR-1972, DANCR/miR‐27a‐3p, HOXA11-AS/miR-124-3p, LINC00339/miR‐152, PITPNA-AS1/miR-448 and EGFR-AS1/miR-145 are examples of ROCK1-regulating axes which contribute in the development of human disorders.

In addition, interactions between non-coding RNAs and ROCK1 has important role in determination of response to a number of drugs such as cisplatin, dexmedetomidine, sevoflurane, curcumin and ginsenoside Rg1. In fact, alterations in the expression levels of ROCK1-interacting non-coding RNAs can affect expression of ROCK1 and induce sensitivity or resistance to these drugs through modulation of cell apoptosis or other fundamental aspects of cell biology. Thus, through modulation of expression of these non-coding RNAs, it is possible to enhance therapeutic effects of these substances.

Based on the above-mentioned evidence, it is clear that ROCK1 has direct or indirect interactions with numerous types of non-coding RNAs constructing a complex network. Identification of elements of this network is an important step for unraveling the molecular pathology of human disorders.
